# From diagnosis to therapy in Duchenne muscular dystrophy

**DOI:** 10.1042/BST20190282

**Published:** 2020-06-29

**Authors:** Arran Babbs, Maria Chatzopoulou, Ben Edwards, Sarah E. Squire, Isabel V.L. Wilkinson, Graham M. Wynne, Angela J. Russell, Kay E. Davies

**Affiliations:** 1 MDUK Oxford Neuromuscular Centre, Department of Physiology, Anatomy and Genetics, University of Oxford, Oxford OX1 3PT, U.K.; 2 Department of Chemistry, Chemistry Research Laboratory, University of Oxford, Oxford OX 3TA, U.K.; 3 Department of Pharmacology, University of Oxford, Mansfield Road, Oxford OX1 3PQ, U.K.

**Keywords:** Dmd, Duchenne muscular dystrophyphy, muscle disease, utrophin

## Abstract

Genetic approaches for the diagnosis and treatment of inherited muscle diseases have advanced rapidly in recent years. Many of the advances have occurred in the treatment of Duchenne muscular dystrophy (DMD), a muscle wasting disease where affected boys are typically wheelchair bound by age 12 years and generally die in their twenties from respiratory failure or cardiomyopathy. Dystrophin is a 421 kD protein which links F-actin to the extracellular matrix via the dystrophin-associated protein complex (DAPC) at the muscle membrane. In the absence of dystrophin, the DAPC is lost, making the muscle membrane more susceptible to contraction-induced injury. The identification of the gene causing DMD in 1986 resulted in improved diagnosis of the disease and the identification of hotspots for mutation. There is currently no effective treatment. However, there are several promising genetic therapeutic approaches at the preclinical stage or in clinical trials including read-through of stop codons, exon skipping, delivery of dystrophin minigenes and the modulation of expression of the dystrophin related protein, utrophin. In spite of significant progress, the problem of targeting all muscles, including diaphragm and heart at sufficiently high levels, remains a challenge. Any therapy also needs to consider the immune response and some treatments are mutation specific and therefore limited to a subgroup of patients. This short review provides a summary of the current status of DMD therapy with a particular focus on those genetic strategies that have been taken to the clinic.

## Introduction

**Figure 1.**
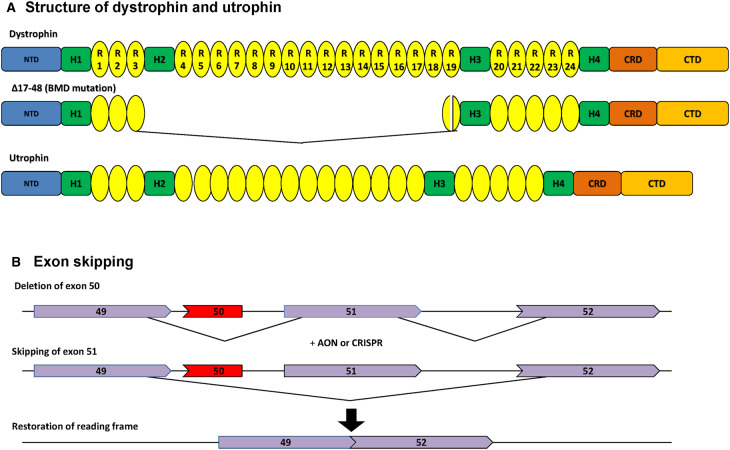
Schematic diagram of the structure of dystrophin, minigenes and utrophin and exon skipping approaches. (**A**) Full length dystrophin comprises N-terminal actin-binding domain (NTD), four hinge domains (H), 24 spectrin-like repeats (R) that form the rod domain, the cysteine rich domain (CRD) which binds the dystroglycoprotein complex and the C-terminal domain (CTD). (**B**) Schematic presentation of exon skipping. Patients with DMD have mutations which disrupt the open reading frame of the dystrophin pre-RNA. In this example, exon 50 is deleted, creating an out-of-frame mRNA and leading to the synthesis of a truncated non-functional unstable dystrophin. An antisense oligonucleotide directed against exon 51 can induce effective skipping of exon 51 and restore the open reading frame, thereby generating an internally deleted but partially functional dystrophin.

Duchenne muscular dystrophy (DMD) is one of the most prevalent neuromuscular disorders and is caused by mutations in the dystrophin gene that result in loss of the key structural protein dystrophin. Dystrophin is an essential component of the dystrophin-associated protein complex (DAPC) which links the actin cytoskeleton to the basal lamina providing stability to the muscle membrane [1]. The N-terminal and C-terminal regions of the dystrophin gene are important for binding F-actin and the DAPC respectively, whilst the central rod domain consists of spectrin-like repeats (Figure 1A). Loss of dystrophin results in chronic inflammation and a vicious cycle of muscle necrosis and regeneration which leads to the eventual replacement of muscle by adipose and connective tissue. This gradual muscle degeneration leads to loss of ambulation in adolescence but ultimately respiratory and cardiac failure, significantly reducing the life expectancy of DMD patients to the second or third decade of life [2]. No effective therapy is currently available and patient management commonly involves the administration of corticosteroids or other anti-inflammatory drugs.

Dystrophin is the largest known gene in humans and consists of 79 exons spread over more than two million base pairs of genomic sequence. Partly because of its large size, the dystrophin locus shows a high level of spontaneous mutation, resulting in the high occurrence of DMD boys in families with no previous history [3,4]. More than sixty per cent of mutations are deletions which vary in size from a few kilobases to more than a megabase [5]. There are a number of challenges in developing therapies for DMD. The mutation heterogeneity complicates the design of disease-modifying therapeutics as many are targeted to a specific mutation and thus are applicable only to a sub-population of patients [2,6,7]. Delivery of dystrophin using gene therapy approaches, or the use of small molecules to increase the levels of the dystrophin related protein, utrophin, are being developed which could be used to treat patients regardless of the underlying dystrophin mutation.

The replacement of the very large 421 kD dystrophin protein is also a challenge and it is estimated that 5–15% of normal dystrophin levels would need to be replaced at the muscle membrane for any therapeutic effect to be observed [8,9].

This review covers the recent progress in the development of genetic approaches to therapy for DMD patients which target the basic defect rather than pharmacological approaches which target the secondary pathology such as inflammation and fibrosis. The latter has been reviewed elsewhere [10]. There are now several genetic approaches which show promise, either correcting the defect at the DNA level using gene editing, at the RNA level through exon skipping, or promoting stop codon read-through at the protein level. Alternatively, the gene product can be replaced using delivery of truncated dystrophin genes using viruses or levels of a surrogate protein can be increased to replace the missing dystrophin. Data from ongoing preclinical studies and clinical trials suggest that effective therapy for DMD patients is on the horizon.

## Stop codon readthrough

Although the vast majority of DMD patients have out-of-frame deletions, ∼11% of patients possess stop codon mutations. Thus a small molecule which could promote readthrough of these nonsense mutations could theoretically produce a full length dystrophin protein (for review see [7]). Clinical trials were initially performed using the antibiotic gentamicin but these were discontinued due to the toxicity of long term treatment. PTC Therapeutics identified a drug, Translarna (ataluren), from a high throughput screen which was reported to show efficient read through in the *mdx* mouse model. However, clinical trials have been disappointing and only low levels of dystrophin are seen after treatment of DMD patients. Translarna is conditionally approved in Europe for ambulatory patients aged two years and older (http://ir.ptcbio.com.releasedetial.cfm?releaseid=863914). FDA approval is still lacking. The antibiotic arbekacin sulfate (NPC-14) also shows premature stop codon readthrough and is currently in Phase 2 clinical trials [6].

## Exon skipping

The vast majority of mutations in the dystrophin gene are clustered in two hot spots, one near the 5′ end and the other clustered around exon 51 [5]. Monaco and colleagues proposed the reading frame rule which stipulates that mutations in exons which removed sections of the gene leaving the reading frame intact would result in a truncated, partially functional protein whose expression resulted in milder disease (for review see [11]). Out of frame deletions would lead to the absence of protein due to nonsense mediated decay and the outcome would be the more severe DMD phenotype. This reading frame rule largely explains the genotype/phenotype correlations observed and has been exploited through exon skipping to convert DMD into a milder Becker muscular dystrophy (BMD). Antisense oligonucleotides (AONs) are chemically synthesized nucleic acids usually 20 bases in length which are designed to hybridize to complementary DNA sequences at exon-intron boundaries or exonic splicing enhancers to promote exon skipping (see Figure 1B). Initially, AONs were developed to promote skipping of exon 51 as this would be applicable to 13–15% of patients [12]. The antisense oligonucleotide backbones most frequently used are the negatively charged 2′-*O*-methyl-phosphorothioate (2′OMeAO) and the charge neutral phosphorodiamidate morpholino oligomers (PMO). The 2′OMeAO targeting the skipping around exon 51 showed very promising data in the *mdx* mouse but failed to demonstrate efficacy in Phase 3 clinical trials. The PMO chemistry has been used in the development of two FDA approved drugs eteplirsen for exon 51 and golodirsen for exon 53 with more morpholinos targeting other exons under development (see www.sarepta.com). However, although the treatment significantly slows the progression of the disease, more efficient exon skipping constructs are needed to provide greater clinical benefit.

The effect of exon skipping lasts 2–3 months necessitating repeated administration and there is a need to target the heart as well as all skeletal muscle. Efforts are underway to improve the targeting efficacy in all muscles by exploring new AON chemistry, tagging the AONs with peptides [13]. Looking to the future, it should be possible to design strategies to skip several exons simultaneously, for example with a deletion of exons 45–55 which would be applicable to ∼60% of patients. This could also be achieved using gene editing strategies (see below).

## Gene therapy

The replacement of the missing dystrophin directly in patients has many challenges, not least because of its large size (427 kD) and the fact that it is encoded by a 14 kb mRNA which is too large to fit into AAV (Adeno-virus associated virus) vectors commonly used as delivery vehicles for muscle as they have a limited capacity of ∼5 kb. Studies of mildly affected BMD patients revealed one individual who had 46% of this coding region missing across the spectrin-like repeat domain (see Figure 1A). This patient had a distant relative who also had the deletion with a mild phenotype and studies of the localization of his truncated minigene showed the truncated protein to be correctly localized at the sarcolemma [14]. Using transgenic *mdx* mice as a test model of function, several groups have designed further deletions to generate microdystrophins which also allow the integration of muscle specific promoters (for reviews see [15–18]). None of the reported microgenes will function as well as the full length dystrophin as they are by necessity a compromise, although encouraging data have been reported in the dog model of the disease [19]. It is difficult to predict how well the corresponding truncated proteins will function in human muscle. The different microgenes currently used in clinical trials reflects this (summarized in [16]). For example, the nNOS (nitric oxide synthase) binding site encoded by exons 16/17 has been reported to be important although several mildly affected patients have been documented who are deleted for these binding sites [20]. Nevertheless, preliminary reports of clinical trials in ambulatory DMD boys treated with microdystrophin AAV therapy have shown very promising results with 96% dystrophin positive fibres (www.sarepta.com), although some adverse effects have been reported in other trials of AAV therapy (for review see [18]).

The challenges of gene therapy include an immune response to the virus and to the microdystrophin, although these can be minimized by transient immunosuppression in treated patients. Any antibody response to micro-dystrophin could be avoided by using constructs based on the structure of the dystrophin related protein, utrophin (see below; [21]) In order to achieve clinical benefit and high levels of dystrophin, high titers of virus need to be administered and there are challenges for production. Because of the turnover of muscle in DMD patients there may be a need for repeat administration. Timing of delivery for the first gene therapy treatment will also be critical as the virus will be lost during muscle growth but administration later in the disease may be limited by the much reduced muscle mass available. Nevertheless much progress has been made.

## AAV-CRISPR/Cas9 genome editing

CRISPR/Cas9 has the potential to revolutionize gene therapy in a wide range of diseases. CRISPR editing has recently reported to show efficacy in mouse and dog models of the disease and this could be applied to multi exon skipping, editing of single base mutations or correction of duplications [22–24]. Although this approach would involve the use of AAV vectors for delivery which has its own challenges detailed above, CRISPR editing has the advantage that it would require a single, rather than multiple, administrations.

## Replacement of dystrophin with a surrogate protein

Utrophin is a structural paralogue of dystrophin and has been proposed as a surrogate for the missing dystrophin in DMD patients (see Figure 1; for review see [10]). Utrophin, unlike dystrophin which is expressed predominantly in muscle with small amounts in brain, is expressed in many tissues. Early in human and mouse development, utrophin is localized alongside dystrophin at the muscle membrane. At birth, dystrophin remains at the sarcolemma but utrophin is only found at the neuromuscular and myotendinous junctions in adult muscle. Both proteins bind a similar complex of proteins at the sarcolemma although there are differences in the isoforms of their binding proteins. In damaged muscle utrophin is re-expressed at the sarcolemma of regenerating fibres, identified by the re-expression of developmental myosin [25]. Utrophin does not bind nNOS nor does it interact in the same way with F-actin [20]. Changes in microtubule organization have been reported which suggests that dystrophin and utrophin function slightly differently in normal muscle [26]. Nevertheless, transgenic mice expressing relatively low levels of utrophin in a uniform manner at the sarcolemma prevent the pathology observed in *mdx* mice. More importantly, a recent report showed that a codon optimized micro-utrophin gene modelled on the micro-dystrophin gene is a compelling surrogate for dystrophin in preventing pathology in the dog model of the disease [21]. In the context of gene therapy, AAV-micro-utrophin may be preferred for the treatment of DMD patients as, unlike with microdystrophin, there will be a much lower risk of an immune response.

The data from utrophin transgenic mice supported the initiation of drug discovery programmes from a number of companies and academic groups [27]. Strategies that increase utrophin protein include increasing the stability of the utrophin mRNA, promoting an oxidative phenotype or using drugs that feed into regulation of transcription of utrophin (for reviews see [10,28]). One drug, ezutromid, developed from transcription reporter assay using a fragment of the utrophin A promoter, was shown to have a positive effect in preventing the pathology in the *mdx* mouse and reduced the number of regenerating fibres [28]. This first-in-class utrophin modulator was developed by the Davies and Russell groups in collaboration with Summit Therapeutics, and progressed from a phenotypic screen through to a Phase 2 proof of concept clinical trial. Promising efficacy and evidence of target engagement was observed in DMD patients after 24 weeks of treatment; however, trial endpoints were not met after 48 weeks. These investigators subsequently generated data that explains the lack of sustained efficacy in the trial [29] and elucidated the molecular mechanism of action (MoA) of ezutromid [30].

The lack of sustained efficacy in the trial may be explained by the extensive metabolism of ezutromid via CYP1A oxidation to predominantly two dihydrodiol metabolites in humans, both of which when tested for utrophin up-regulation showed substantially reduced potency or no activity [29]. Moreover, repeated dosing of ezutromid leads to a reduction in exposure in both healthy volunteers (∼20% reduction after 10 days) and markedly in DMD patients (∼60% reduction after 10 days) [31] through induction of CYP1A [29,32,33]. This means early in the trial, the patients are exposed to sufficient ezutromid to achieve target engagement and utrophin up-regulation, but at later timepoints exposure is insufficient leading ultimately to loss of utrophin from the membrane and loss of efficacy. Turnover of dystrophin at the muscle membrane is known to be slow and, due to their structural similarities, it would be anticipated that utrophin is also maintained at the membrane for several months. This could account for the observed positive clinical effect followed by a lack of sustained efficacy in the clinical trial.

**Figure 2.**
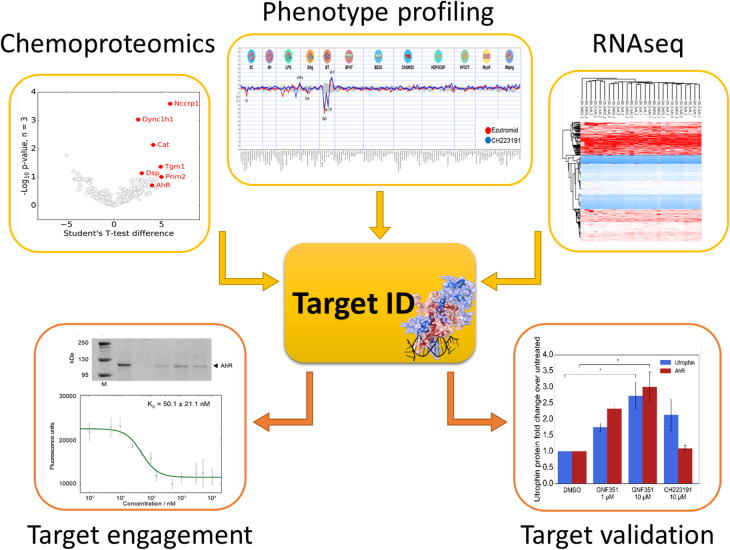
Target deconvolution studies for the utrophin modulator ezutromid. Input from chemoproteomics, phenotype profiling and RNA seq converged on the aryl hydrocarbon receptor, which was further confirmed with target engagement and target validation studies, (based on figures from [30]).

**Figure 3.**
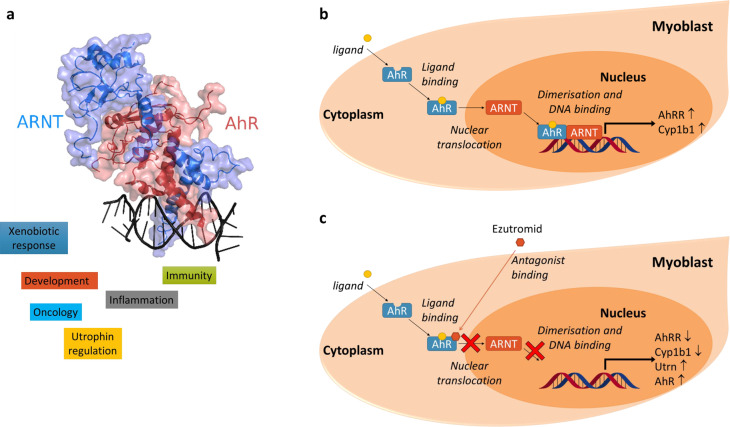
Regulation of biological processes via AhR and the relationship with utrophin. (**a**) Crystal structure of truncated AhR (red) and AhR nuclear translocator (ARNT, blue) heterodimer in complex with DNA, PDB deposit: 5v0l. (**b**) Treatment with AhR ligands results in nuclear translocation, dimerization with ARNT, binding to DNA and transcription of XRE/DRE elements such as AhRR and Cyp1a1/Cyp1b1. (**c**) Treatment of myoblasts with AhR antagonists like ezutromid down-regulates nuclear translocation of AhR by inhibiting dimerisation with ARNT, and/or DNA binding. This results in down-regulating the transcription of AhR responsive genes, and at the same time increases utrophin and AhR transcription and translation.

Using a combination of chemical proteomics and phenotypic profiling, the investigators went on to demonstrate that the aryl hydrocarbon receptor (AhR) is a molecular target of ezutromid [30]. They have generated several lines of evidence to show that ezutromid binds to AhR *in vitro* and in muscle cells and functions as an AhR antagonist (see Figure 2). Importantly they have also demonstrated that other structurally distinct AhR antagonists also increase utrophin protein levels in human DMD muscle cells, revealing AhR as a potential therapeutic target for DMD [30]. As AhR is a pleiotropic transcription factor involved in regulating development, immunity and the xenobiotic response (see Figure 3), it is important to maintain the desired AhR-mediated activity while avoiding potentially toxic effects. For example, activation of pro-inflammatory acute phase response gene expression is induced by some AhR antagonists but not all [34,35]. In addition, some but not all AhR antagonists lead to an increase in AhR expression, the consequences of which are unknown for long-term administration in humans. Thus far, a mechanism by which AhR antagonism leads to utrophin up-regulation has not yet been established. However, prototypical AhR agonist TCDD is known to decrease activity and levels of transcriptional co-activator peroxisome proliferator-activated receptor-γ co-activator-1α (PGC1α) [36], which stimulates utrophin expression at neuromuscular junctions [37]. Treatment with AhR antagonists may increase utrophin by stabilizing active PGC1α.

AhR is seeing a resurgence of interest in a range of other disease indications and early phase clinical trials are underway with AhR antagonists in oncology indications and rheumatoid arthritis. Yet, few AhR antagonists, such as ezutromid have been described [38–40]. Exploitation of this pathway to increase utrophin level thus represents an exciting potential opportunity to benefit DMD patients.

One of the concerns about increasing levels of utrophin in the more mildly affected patients has been that the utrophin might displace truncated dystrophin at the membrane. However, a recent study has shown that dystrophin and utrophin can be co-localised at the muscle membrane suggesting the utrophin up-regulation could be used to treat BMD patients who express only small amounts of dystrophin or truncated dystrophin [41]. Furthermore, increasing utrophin with a small molecule could be used together with any of the other genetic approaches, none of which on their own can restore normal muscle function.

## Concluding remarks

The dystrophin gene was identified more than 30 years ago. Only now is an effective therapy on the horizon. The genetic tool box that can be applied to develop treatment for this progressive muscular dystrophy promises to greatly improve the quality of life of DMD patients and extend their lives within the next 5–10 years. Only exon skipping and stop codon readthrough have been approved so far but these do not have a major clinical effect although they do slow progression of the disease. The field is eagerly awaiting the results of the ongoing clinical trials using improved current and other approaches.

## Perspectives

- DMD is one of the most common muscular dystrophies where there is a high unmet clinical need as there is a high new mutation rate. No effective genetic therapy has been approved and pharmacological approaches only slow down the disease progression.
- Genetic approaches to therapy such as stop codon readthrough, exon skipping, gene therapy or increased expression of a surrogate protein to replace the missing dystrophin are making excellent progress.
- Gene therapy is showing great promise but there are challenges of repeat administration because of the potential immune response to the virus and the age at which the therapy could be administered for greatest effect. The application of a combination of the therapies available may have the greatest impact on improving the quality of life for DMD patients.

## Open Access

Open access for this article was enabled by the participation of University of Oxford in an all-inclusive *Read & Publish* pilot with Portland Press and the Biochemical Society under a transformative agreement with JISC.

## Abbreviations

AAV: adeno-virus associated virus
AhR: aryl hydrocarbon receptor
AONs: antisense oligonucleotides
BMD: Becker muscular dystrophy
DAPC: dystrophin-associated protein complex
DMD: Duchenne muscular dystrophy
nNOS: nitric oxide synthase
PMO: phosphorodiamidate morpholino oligomers
